# The Interplay between the Bone and the Immune System

**DOI:** 10.1155/2013/720504

**Published:** 2013-07-14

**Authors:** Giorgio Mori, Patrizia D'Amelio, Roberta Faccio, Giacomina Brunetti

**Affiliations:** ^1^Department of Clinical and Experimental Medicine, University of Foggia, 71100 Foggia, Italy; ^2^Department of Medical Sciences, University of Torino, 10126 Torino, Italy; ^3^Department of Orthopedics, Washington University School of Medicine, St. Louis, MO 63110, USA; ^4^Department of Basic Medical Sciences, Neurosciences and Sense Organs, Section of Human Anatomy and Histology, University of Bari, Piazza Giulio Cesare, 11, 70124 Bari, Italy

## Abstract

In the last two decades, numerous scientists have highlighted the interactions between bone and immune cells as well as their overlapping regulatory mechanisms. For example, osteoclasts, the bone-resorbing cells, are derived from the same myeloid precursor cells that give rise to macrophages and myeloid dendritic cells. On the other hand, osteoblasts, the bone-forming cells, regulate hematopoietic stem cell niches from which all blood and immune cells are derived. Furthermore, many of the soluble mediators of immune cells, including cytokines and growth factors, regulate the activities of osteoblasts and osteoclasts. This increased recognition of the complex interactions between the immune system and bone led to the development of the interdisciplinary osteoimmunology field. Research in this field has great potential to provide a better understanding of the pathogenesis of several diseases affecting both the bone and immune systems, thus providing the molecular basis for novel therapeutic strategies. In these review, we reported the latest findings about the reciprocal regulation of bone and immune cells.

## 1. Introduction

Bone remodelling, a coordinated process between formation and degradation of bone, respectively managed by osteoblasts (OBs) and osteoclasts (OCs), ensures the bone homeostasis. In physiological conditions, canonical OC formation requires macrophage colony-stimulating factor (MCSF) and receptor activator factor of nuclear factor kB ligand (RANKL) [1], which act on cells of the monocyte-macrophage lineage, inducing their fusion to form polynucleated active resorbing cells. However, a number of other cytokines and growth factors are known either to substitute these two molecules inducing a noncanonical OC formation or to act indirectly on osteoclastogenesis promoting RANKL release from other cells [1]. Physiologically, osteoclastogenesis is sustained by OBs, cells arising from the bone marrow stromal cells (BMSCs) which following the activation of different pathways and specific transcription factors, such as Cbfa1/Runx2, differentiate in mature cells producing bone matrix [2]. Consistently, OB activity can be also regulated by OCs. In the attempt to understand the mechanisms regulating bone remodelling, it has been found that skeletal homeostasis is dynamically influenced by the immune system, and lymphocyte- or macrophage-derived cytokines are among the most potent mediators of osteoimmunological regulation [3, 4]. Thus, in this review we will describe osteoclastogenesis, osteoblastogenesis, and the role of immune system in regulating the activity of bone cells.

## 2. Osteoclastogenesis

OCs are formed by the attraction of myelomonocytic precursors to the resorption site, followed by their fusion, and attachment of the subsequent multinucleated cell to the bone surface. This process requires the activation of critical intracellular pathway as well as specific cytokines, primarily M-CSF and RANKL, but also TNF-*α*, IL-1, IL-7, IL-17, IL-23, IL-6, TGF*β*, and IFN*γ*. Most of these molecules are also involved in the regulation of immune system and this may explain some of the relationship between immune and bone cells [5].

### 2.1. M-CSF

M-CSF is a homodimeric glycoprotein, produced by OBs and bone marrow stromal cells, that binds to high-affinity receptors (c-fms) expressed on cells of the monocyte/macrophage lineage. Homozygous disruption of M-CSF coding sequences in osteopetrotic (op/op) mice severely impairs production of macrophage populations underlying the importance of M-CSF for their development [6]. M-CSF induces the proliferation of OC precursors, their differentiation and increases the survival of mature OCs [7]; OC formation occurs when monocytes are costimulated by the essential osteoclastogenic factors M-CSF and RANKL.

### 2.2. RANKL/RANK/OPG System

A central role in OC biology is played by the receptor activator of NF-kB ligand (RANKL), that is essential for osteoclastogenesis and bone resorption [8]. Mice and humans deficient in the RANKL gene completely lack OC and exhibit variable forms of osteopetrosis. RANKL has also been implicated in regulation of immune response and in arterial wall calcification [5, 9]. The functional receptor for RANKL, RANK, is encoded by a tumour necrosis factor receptor (TNFR) superfamily gene (TNFGS11A) and is expressed on OC precursors. Mice lacking TNFGS11A have a profound defect in bone resorption and in the development of cartilaginous growth plates. One of the key steps upon activation of the RANK pathway is the binding of TNFR-associated cytoplasmic factors (TRAFs) to specific domains within the cytoplasmic domain of RANK. The TRAF family proteins are cytoplasmic adapter proteins involved in the mediation of several cytokine-signalling pathways. Different members of the family activate different transcriptional pathways: TRAF2, 5, and 6 are involved in the activation of NF-kB through IkB kinase (IKK) activation and AP-1 through activation of mitogen-activated protein kinases (MAPKs), including Jun-N-terminal kinase (JNK), p38, and extracellular signal-regulated kinase (ERK). Moreover, TRAF6 functions as a ubiquitin ligase, which catalyzes the formation of a polyUb chain. This leads to the activation of IKK and JNK through a proteasome-independent mechanism [10]. 

RANKL/RANK signalling promotes the differentiation of OC precursors into mature multinucleated OCs, stimulates their capacity to resorb bone, and decreases OC apoptosis. RANKL is present as both a transmembrane molecule and a secreted form; its interaction with RANK is opposed by osteoprotegerin (OPG), a neutralizing soluble decoy receptor, produced by marrow stromal cells and OBs [11]. The unbalance between RANKL and OPG has been indicated as the pivotal mechanism responsible for bone loss in case of estrogen deficiency [12], inflammation [13], and cancer-induced bone loss [14].

### 2.3. TNF-*α*

TNF-*α* enhances OC formation by upregulating stromal cells production of RANKL and M-CSF and by augmenting the responsiveness of OCs precursors to RANKL. TNF directly induces marrow precursor differentiation into OCs, although according to some studies it is not osteoclastogenetic in cells not previously primed by RANKL. The ability of TNF to increase the osteoclastogenic activity of RANKL is due to synergistic interactions at the level of NFkB and AP-1 signalling. In addition, TNF and RANKL synergistically upregulate RANK expression. *In vivo* blockade of TNF in postmenopausal osteoporosis reduces bone resorption [15]; this suggests that TNF-*α* increase could be one of the mechanisms responsible for postmenopausal bone loss. TNF is mainly produced by activated T cells and it is also involved in inflammation and cancer induced bone loss both systemically and locally.

### 2.4. IL-1

IL-1 plays an important role in bone loss induced by estrogen deficiency; its level increases after menopause and is reversed by estrogen replacement. Bone loss does not occur after ovariectomy in mice deficient in receptors for IL-1, and treatment with IL-1 receptor antagonist decreases OC formation and activity. A recent study demonstrates that the blockade of IL-1 reduces bone resorption in postmenopausal osteoporosis [15]. IL-1 acts by increasing RANKL expression by bone marrow stromal cells and directly targets OC precursors, promoting OC differentiation in the presence of permissive levels of RANKL. The effect of TNF-*α* on osteoclastogenesis is upregulated by IL-1.

### 2.5. IL-7

IL-7 is known for its ability to stimulate T and B cell number and the reaction to antigenic stimuli. Recently, a role for IL-7 has also been postulated in bone remodelling [16, 17]. We have demonstrated that IL-7 promotes osteoclastogenesis by upregulating T and B cell-derived RANKL [17] and that the production of IL-7 is downregulated by estrogen. 

In humans it has been suggested that IL-7 is osteoclastogenic in psoriatic arthritis and in solid tumors, also in healthy volunteer the expression of IL-7 receptor on T lymphocytes correlates with their ability to induce osteoclastogenesis from human monocytes.

### 2.6. IL-17, IL-23, and IL-27

IL-17 family members are mainly expressed by a type of human T helper cell (Th17) [18]. It is now believed that this cytokine plays a crucial role in inflammation and the development of autoimmune diseases such as rheumatoid arthritis; however, its mechanism of action in the development of bone erosions, especially in relation to other known key cytokines such as IL-1, TNF-*α*, and RANKL, remains unclear. Recently, IL-17 has been suggested to be involved in the upregulation of OC formation in inflammation by increasing the release of RANKL, which may synergise with IL-1 and TNF [19]. One of the stimuli to IL-17 synthesis is IL-23 produced by activated dendritic cells and macrophages. IL-23 drives the T helper 1 response and is a implicated in autoimmune diseases; hence; it has been suggested that the IL-23/IL-17 axis is critical for controlling inflammatory bone loss. However, in contrast to IL-17-deficient mice, IL-23 knockout mice were completely protected from bone and joint destruction in the collagen-induced arthritis model, indicating that the IL-23-induced bone loss may not be entirely mediated by IL-17 and raising the question whether IL-23 can directly stimulate OCs. Recent work supports this hypothesis suggesting that IL-23 promotes OC formation [20]. Other recent *in vivo* studies suggest that IL-23 inhibits OC formation via T cells [21]. In physiological conditions (unlike inflammatory conditions), IL-23 favours higher bone mass in long bones by limiting resorption of immature bone forming below the growth plate [21]. These contrasting data suggest different roles of this cytokine in the control of physiological or inflammatory bone turnover. Recently, Interleukin-27 (IL-27) raises investigator attentions as an antiosteoclastogenic cytokine [22, 23]. In particular, it suppresses osteoclastogenesis both through a direct effect on OCs and an indirect action on T helper cell subsets [22–26]. On OC precursors IL-27 decreases the ability to differentiate into fully mature resorbing cells, by abrogating RANKL-mediated induction of NFATc1 and suppressing proximal RANK signalling [22, 23]. On T helper (Th) subsets, it favours the differentiation of T cells in Th1 cells, promotes the differentiation of regulatory T cells, and decreases the differentiation of Th 17 cells, resulting in osteoclastogenesis inhibition in inflammatory condition [24–26].

### 2.7. IL-6

Activation of the signalling pathway mediated by glycoprotein (gp) 130 by IL-6 and its soluble receptor has been regarded as a pivotal mechanism for the regulation of osteoclastogenesis [27]. Nevertheless, in IL-6 knockout mice (IL6KO), as well as in gp 130-deficient mice, no decrease in OC formation and function was found. These data may suggest that IL-6 is not essential for bone resorption. However, IL6KO mice were protected against ovariectomy-induced bone loss, and this finding, together with the observation of increased level of IL-6 after menopause in women, may suggest a peculiar role for IL-6 in bone loss due to estrogen deprivation. IL-6 was also shown to be involved in other diseases associated with accelerated bone turnover such as Paget's disease of bone, multiple myeloma, rheumatoid arthritis and renal osteodystrophy.

### 2.8. IFN*γ*

The effect of IFN*γ* on OC formation and activity is controversial. IFN*γ* behaves like an antiosteoclastogenic cytokine *in vitro* [28], *in vivo* in nude mice [29] and in a knockout models in which the onset of collagen-induced arthritis is more rapid, as compared with wild-type controls. These data are not confirmed by studies in humans and in experimental models of diseases that indicate an increased level of IFN*γ* during estrogen deficiency.

In humans IFN*γ* is positively correlated with bone erosions in leprosy and rheumatoid arthritis. Data from randomized controlled trials have shown that IFN*γ* does not prevent bone loss in rheumatoid arthritis. The use of IFN*γ* in humans has been suggested to employ IFN*γ* for the treatment of osteopetrosis, in which condition IFN*γ* is able to restore bone resorption.

Taken together, the data in humans suggest that, in some conditions, IFN*γ* stimulates bone resorption. These discrepancies could be explained by the fact that IFN*γ* directly blocks OC formation targeting maturing OC and induces antigen presentation and thus T cell activation *in vivo*. Therefore, when IFN*γ* levels are increased *in vivo*, activated T cells secrete proosteoclastogenic factors and this activity offsets its antiosteoclastogenic effect.

### 2.9. TGF*β*

TGF*β* plays a complex role in osteoclastogenesis. It has wide ranging effects and it has been suggested that it may play a pivotal role in the growing skeleton contributing to the coupling between OB and OC [30]. Three isoforms of TGF*β* have been described (TGF*β*1–3), which all interact with the same receptor complex. TGF*β*1 is mainly expressed in lymphoid organs and in serum. Conversely, TGF*β*2 and TGF*β*3 are predominantly expressed in mesenchymal tissues and bone. TGF*β* is produced by many cell types, including bone marrow cells, OBs, and stromal cells and is secreted in a latent form that must be activated to mediate its effects. Although several mechanisms of activation *in vivo* have been proposed, the precise mechanism of this process is not known. Both *in vitro* and *in vivo* studies have shown that TGF*β*1–3 have complex effects on bone. They stimulate or repress proliferation or formation of OBs and Ocs, depending on cell types and culture conditions used. Mice with OB-specific overexpression of TGF*β*2 develop high-turnover osteoporosis [31].

TGF*β* has also been implicated in the pathogenesis of ovariectomy-induced bone loss because local injection of TGF*β*1 and TGF*β*2 prevent bone loss at the site of the injection in ovariectomy rats. Furthermore, estrogen is known to upregulate the expression of TGF*β* in murine OBs, bone extracts and bone marrow cells and long-term *in vivo* estrogen treatment has been shown to increase serum TGF*β*1 and TGF*β*2 levels in humans. Latent TGF*β* is abundantly present in the bone matrix and is released and activated during bone resorption, and it feeds back to modulate OB and OC activity. In particular TGF*β* is believed to induce OCs apoptosis that follows bone resorption *in vivo* [31].

## 3. Osteoblastogenesis

OBs differentiate from mesenchymal stem cells (MSCs), sharing their origin with the cells of connective tissues such as fibroblasts adipocytes and chondrocyte; they represent only 5% of total resident cells that are mainly constituted by osteocytes. OBs attend the crucial function of building the bone [2]. During embryonic development OBs originate from local mesenchyme of sclerotome and, in adults, from MSCs or bone marrow stromal cell. Recent works have demonstrated *in vitro* that also human postnatal mesenchymal cells from dental tissues could originate mature OBs [32–36]. 

In response to specific stimuli, these precursors commit to osteogenic lineage and differentiate before in pre-OBs, then in lining cells, and finally in mature OBs. Osteoblastogenesis is defined by several phases: lineage commitment, proliferative expansion, synthesis and mineralization of extracellular matrix, and establishment of osteocyte. All these stages are characterized by sequentially expressed genes that lead to the expression of specific proteins that often are used as specific OB markers. These proteins are collagenic and constitutive as collagen I, enzymatic as alkaline phosphates (ALP), mainly with adhesive function as bone sialoprotein, osteonectin, osteopontin, with different metabolic functions as osteocalcin, and rich in carbohydrates as biglycan and decorin.

Mature OBs, the bone-forming cells, are basophilic, mononuclear, polygonal, and able to secrete all the component of bone matrix. OBs involved in matrix deposition show the typical features of cells acting in an intense protein synthesis: a corrugated cell membrane, a well-developed rough endoplasmic reticulum, with dilated cisternae, a prominent Golgi complex, several free ribosomes, and an euchromatic nucleus with a voluminous nucleolus. Usually OBs are found on bone matrix they are secreting, close to each other, assuming the typical aspect of the lining cells. Once they become surrounded by the matrix, they gradually lose the basophilia, emit cellular process, extending into the newly deposited matrix called osteoid, after matrix mineralization OBs reduce their size and transform in osteocytes. OB cytoplasm contains PAS-positive granules holding the precursors of bone matrix glycoproteins. The plasma membrane of OBs is particularly enriched in ALP, an enzyme which is the characteristic OB marker [37]. 

OBs form tight junctions with adjacent cells, assuming an epithelioid morphology and start matrix deposition secreting the organic component [38].

OBs perform the matrix mineralization process secreting hydroxyapatite crystals surrounded by plasma membrane: the matrix vesicles. The process forming Ca_3_ (PO_4_)_2_ (tricalcium phosphate) in vesicles is not yet completely known: it involves calcium-binding proteins such as calbindin D9k, BSPII, calcium-binding phospholipids, phosphatidylserine, calcium channel-forming annexins [39, 40], and phosphate transporters and enzymes [41]. Once the accumulation of calcium and phosphate overcomes the point of solubility, hydroxyapatite crystals form within matrix vesicles.

The next event in the mineralization process is the hydroxyapatite crystals extravesicle development that fills the intercollagen fibrils spaces; when they assemble in the first stable form named “critical nucleus,” the crystal growth becomes faster increasing its size by ions addition.

The correct hydroxyapatite crystals growth requires typical OB marker ALP action: ALP hydrolyzes inorganic pyrophosphate (PPi) forming two inorganic phosphate (Pi) molecules that are assembled in hydroxyapatite crystals [42].

OBs are the most important cells regulating bone remodelling balance. The OB expresses PTH receptor whose binding to the hormone can activate OC activity increasing serum calcium levels [43, 44]. Thus pre-OBs, OBs, and stromal cells produce two factors acting on OC: the RANKL and OPG [45]. RANKL stimulates osteoclastogenesis and mature OCs activity, OPG, vice versa binding to the same RANKL receptor RANK as competitive ligand, inhibits both these actions.

*Mechanical Loading*. Mechanical loading has prominent influences on OBs and bone remodelling. Disuse or lack of loading causes an acceleration of bone turnover, with OC resorption dominating OB formation with the result of a substantial bone mass loss [46]. This type of bone loss has also been observed in astronauts who spend extended periods of time in the microgravity environment of a space station or shuttle. Lining cells are ubiquitous on bone surface and they contain gap junction connections to osteocytes and OBs. Thus mechanical loads can propagate from osteocytes to bone lining cells and vice versa. Osteocytes do not respond directly to mechanical strain (deformation) of bone tissue but sense the extracellular fluid flow variation caused by loading. Bone surfaces are mostly covered with lining cells that can potentially differentiate into mature OBs two days after a mechanical stimulus [47]; within other two days new bone matrix deposition can be observed [48]. Moreover loading stimulates OB precursor proliferation and differentiation. Mechanical forces also reduce programmed cell death in osteocytes [49] and in active OBs [50]; furthermore loading may extend the rate of bone mineral matrix deposition for each OB.

### 3.1. Transcriptional Factors Regulating Osteoblast Differentiation

The differentiation of OBs from MSCs, which can also originate fibroblasts, chondrocytes, myoblasts, adipocytes, and tendon cells, requires the activity of specific transcription factors that are expressed at distinct time points during the differentiation process, thereby defining various developmental stages of the osteoblastogenesis.

*Runt Domain-Containing Transcription Factor (Runx2)*, also named cbfa1, is a master gene for OB differentiation. Levels of Runx2 gradually increase in subsequent stages of OB differentiation, with maximum expression observed in the mature OBs. Homozygous deletion of Runx2 in mice results in a complete lack of OBs [51]. Runx2 is both necessary and sufficient for mesenchymal cell differentiation towards the OB lineage [52]. It was demonstrated that Runx2 controls OB lineage cells by binding to the Runx regulatory element in promoters of osteoblastogenesis genes. Runx2 target genes include both genes expressed by immature and differentiated OBs, such as TGF-*β* receptor, ALP, collagen type I, OPN, OSTC, vitamin D receptor, BSP, and OPN [53]. Thus Runx2 is necessary for both OB differentiation and activity [54].

*Osterix* is another DNA-binding transcription factor that is absolutely required for OB differentiation. Osterix/SP7 is a member of the zinc-finger-containing SP family and is largely expressed throughout OB differentiation. Genetic inactivation of Osterix in mice results in absence of mineralized bone matrix, defective OBs and perinatal lethality [55]. Similarly to Runx2, forced expression of Osterix in nonbone cells, promote expression of both early and late marker genes of OBs. However, molecular and genetic studies revealed that Runx2 is expressed in mesenchymal tissues of Osterix-null mice [55]. Thus, Osterix acts downstream of Runx2 in the transcriptional cascade of OB differentiation. Consistently, Osterix expression is positively regulated by direct binding of Runx2 to a responsive element in the promoter of the Osterix gene.

*Activating Transcription Factor-4* (*ATF4*), a member of the basic Leu zipper family of transcription factors, has important roles in the mature stage of OB differentiation. Misregulation of ATF4 activity has been linked with the skeletal abnormalities seen in human patients with the Coffin-Lowry syndrome and neurofibromatosis type I [56]. ATF4 may function in OB lineage cells through two distinct mechanisms. First, it directly regulates the expression of osteocalcin and RANKL [56]. Second, ATF4 promotes efficient amino acid import to ensure proper protein synthesis by OBs [56].

### 3.2. Regulation of Osteoblast Differentiation by Secreted Molecules

Bone cells, as well as many other cells present in bone marrow compartment, produce numerous growth factors and cytokines that act on OBs in both autocrine and paracrine ways to control cell proliferation, differentiation, and survival. These secreted factors and signalling pathways either promote or suppress the expression of transcription factors essential for OB differentiation.

*TGF-*β*1* has a variety of widely recognized roles in bone formation. For example, TGF-*β*1 enhances OB proliferation [57], blocks apoptosis of OBs [58], and also recruits osteoblastic precursors or matrix-producing OBs to the site through chemotactic attraction [59]. In addition, TGF-*β*1 enhances the production of extracellular bone matrix protein by OBs in the early stages of OB differentiation [60]. On the other hand, TGF-*β*1 inhibits the later phase of OB proliferation and mineralization [61]. It has been previously reported that both TGF-*β* receptor I and receptor II expression in murine, rat, and human OBs were decreased during OB differentiation, which may imply that OBs are less sensitive to TGF-*β*1 in the late phase of their differentiation [62]. The later stages are positively regulated by bone matrix proteins (BMP), which are members of the TGF-*β* superfamily [63]. Therefore, TGF-*β*1 cooperates with BMP to regulate the differentiation of OBs. 

Moreover several reports indicate that Runx2 is regulated by TGF-*β*1 and BMP-2. In the initial phase of osteoblastic differentiation (differentiation of MSCs to OB progenitor cells), Runx2 inhibited differentiation of MSCs to types of cells other than OBs, which required coordinated action between Runx2 and BMP2-induced Smad5 [64]. In the second phase of their differentiation (from OB progenitor cells to OBs), TGF-*β*1 induced the expression of Runx2, which crosstalks with beta-catenin signaling to promote differentiation [59]. However, in the final differentiation stages of OBs (mature OBs), TGF-*β*1 opposes BMP-2 actions [58]. Smad3, activated by TGF-*β*1, physically interacts with Runx2 at Runx2-responsive elements and suppresses the expression of Runx2.

*Bone Morphogenetic Proteins (BMPs)*, belonging to the TGF-*β* superfamily, were originally identified as the active components in bone extracts capable of inducing ectopic bone formation. BMPs are expressed in bone, are required for skeletal development and maintenance of adult bone homeostasis, and play an important role in fracture healing [62]. Genetic studies have demonstrated an important roles for BMP2 and BMP4 in promoting OB differentiation and function [63]. Recent studies have also shown that BMP3 inhibits the signal transduced by BMP2 or BMP4 [64] working as a negative regulator of OB differentiation.

*The Wingless (Wnt) Family of Glycoproteins* has recently emerged as central regulators of bone mass [65–77]. Upon engaging various membrane receptors, Wnt ligands activate numerous intracellular pathways that are either dependent on or independent of *β*-catenin [65–67, 75, 76]. In the *β*-catenin-dependent signalling, Wnt binds to the Frizzled receptors and their co-receptors low-density lipoprotein receptor-related protein 5 or 6 (LRP5/6) to stabilize cytosolic *β*-catenin that enters the nucleus and stimulates the transcription of Wnt target genes such as Runx2, Osterix, Fra-1, and Fra-2 [70]. Thus activation of the canonical pathway promotes the differentiation of OB progenitor cells into mature OBs. Wnt signalling is tightly regulated by a delicate balance of extracellular agonists and antagonists. There are different antagonists, such as soluble Frizzled-related proteins (sFRPs) which inhibit Wnt signaling by binding to and sequestering Wnt ligands, and others belonging to the Dickkopf (DKK) family members or the SOST gene product (sclerostin) that bind to and sequester the Wnt coreceptors LRP5/6. In humans, receptor mutations that render Wnt signal constitutively active result in a generalized increase in bone mass [71]. Loss-of-function mutations in the gene encoding the Wnt coreceptor LRP5 cause osteoporosis-pseudoglioma syndrome [72], a form of juvenile-onset osteoporosis. Conversely, mutations in LRP5, that inhibit the interaction between the co-receptor and DKK1 or sclerostin, cause high bone mass syndrome [71, 73, 74]. In addition, loss-of-function or loss-of-expression mutations in SOST, result in the bone-thickening diseases sclerosteosis or van Buchem disease, respectively [73, 74, 77]. *β*-catenin-independent Wnt signalling has also been implicated in promoting OB differentiation [75]. In particular, Wnt 5A is thought to promote OB differentiation by inhibiting the activation of adipogenic genes [76]. Thus both *β*-catenin-dependent and *β*-catenin-independent Wnt signalling are able to control differentiation of OB progenitor into mature OBs.

*Fibroblast Growth Factors (FGFs)* are a large family of proteins (23 different ligands) that transduce their signal through one of the four FGF receptors (FGFR). FGFs initiate condensation of the mesenchyme and proliferation of progenitor cells. In particular, FGF2 is important for pre-OB proliferation and maturation [78], while FGF18 is essential in mature OB formation [79]. 

*TRAIL* is a cytotoxic protein inducing apoptosis, upon binding to death domain-containing receptors DR4 and DR5; its activity can be modulated by association with two membrane-bound decoy receptors, namely, DcR1 and DcR2, lacking functional death domains and conferring TRAIL resistance on expressing cell [80, 81]. Thus the sensitiveness of TRAIL-induced apoptosis is determined by the ratio of death and decoy receptor. OBs express TRAIL receptors, but in normal conditions they are less sensitive to its apoptotic effects [82]; however in inflammatory conditions as periodontal disease TRAIL profoundly can affect OB stimulating their apoptosis, impairing bone remodelling because of a decreased bone formation [83]. 

## 4. Immune and Bone Cell Relationship

### 4.1. T Cells

T cells are critical mediators of the adaptive immune response. These lymphocytes may be subdivided into major classes according to the subunits which form the T cell receptor (TCR). T cells express either an *αβ* or *γδ* TCR on the cell surface, and these receptors are responsible for recognizing a diverse range of antigens [84]. Most T lymphocytes are *αβ* T cells, a lineage which express either the CD4 or CD8 marker. By contrast, the majority of *γδ* T cells lack expression of CD4 and CD8 and their function is poorly understood [85]. Another small subset of T cells is known as natural killer T (NKT) cells [86]. Although small in number, NKT cells can produce large amounts of cytokines and have been implicated in a variety of immune responses including autoimmunity, graft rejection, and responses to pathogens [87]. Together with their prominent role on immune response T cells also can affect bone remodelling. In particular, under basal conditions T cells are not considered a significant source of RANKL, and T-cell-deficient nude mice do not show evidence of diminished RANKL mRNA in their BM [87]. The bone protective role of resting T cells was, however, clearly demonstrated by the finding that T-cell-deficient mice have a significant increased in basal OC number and reduced bone density as compared to controls [87, 88]. Moreover, resting T cells have been shown to blunt OC formation *in vitro* [89] and may contribute to dampen bone resorption *in vivo* [87]. In fact, depletion of CD4^+^ and CD8^+^ T lymphocytes in mice *in vivo* enhances OC formation by a mechanism involving the complete suppression of osteoprotegerin production by B cells [90]. Providing further support to this hypothesis others have found that 1,25 dihydroxyvitamin D3 was a more potent inducer of OC formation in cultures of BM from T-cell-depleted mice than from control mice [90]. In contrast, it is well established that infection and inflammation lead to T cell activation and T cell production of osteoclastogenic cytokines such as RANKL and TNF*α* (TNF). Indeed activated T cells have been implicated in the bone loss in inflammation, autoimmune disorders [91, 92], periodontitis [93, 94], cancer [95], and osteoporosis models [96, 97]. However, distinct function should be attributed to the different T cell subsets on bone remodelling.

### 4.2. CD4^+^ T Cells

These cells represent one of the main components of the adaptive immune response. After antigenic stimulation, naïve CD4^+^ T cells proliferate and may differentiate into distinct effector subsets, which have been classically divided, on the basis of their cytokine production profiles, into Th1 and Th2 cells [98]. Th1 cells are characterized by the secretion of IFN-*γ*, IL-2, IL-12, tumor necrosis factor (TNF)-*α*, and TNF-*β* and are involved in the eradication of intracellular pathogens. Conversely, Th2 cells, characterized by secretion of IL-4, IL-5, IL-6, IL-9, and IL-13, which are potent activators of B cells, are involved in the elimination of extracellular microorganisms and parasitic infections and are also responsible for allergic disorders [99, 100]. In a comprehensive study by Sato et al., Th1 and Th2 cells were both shown to inhibit OC formation through their canonical cytokines IFN-*γ* and IL-4, respectively [101]. More recently, two new subsets of CD4^+^ T cells have been characterized; on the one hand, the Th17 subset, which follows different polarizing conditions and displays different functional activities from those of Th1 and Th2 cells [102, 103] and, on the other hand, the regulatory T (Treg) cell subset, which can be defined based on expression of CD25 and the transcription factor FoxP3 and are critical in the prevention of autoimmune disease [104, 105]. Of these, Th17 T cells have been suggested to be the osteoclastogenic T cells.

#### 4.2.1. Th17

Th17 cells are produced when naïve T cells are activated by TGF*β* and IL-6 in the mouse or TGF*β* and inflammatory stimuli in humans. The resulting clonal memory T cell population will be instructed to produce the Th17 signature cytokines IL-17A, IL-17F, IL-22, and IL-26 [106]. A subset of Th17 cells also produce small amounts of IFN*γ*, which *in vitro* moderate the osteoclastogenic activity of Th17 [107]. The cytokine repertoires of specific Th17 subsets depend on master differentiation factors present in the microenvironment during initial antigen recognition [106]. Thus, Th17 cells promote osteoclastogenesis mostly through production of IL-17, which as known acts on OC precursors to induce RANK [108, 109] and induces RANKL expression in stromal cells and OBs. However, Th17 cells produce additional cytokines relevant for bone, including RANKL and TNF [108]. It should be noted that the effect of IL-17 is not limited to this direct effect on the osteoclastogenesis-supporting cells. IL-17 facilitates local inflammation by recruiting and activating immune cells, which leads to an abundance of inflammatory cytokines such as TNF-alpha and IL-1 [108]. The inflammatory cytokines enhance RANKL expression on osteoclastogenesis-supporting cells and activate OC precursor cells by synergizing with RANKL signaling. A prominent role for Th17 has been demonstrated in bone diseases, such as multiple myeloma and arthritis [110, 111].

#### 4.2.2. CD4 T Reg

The anti-inflammatory Treg inhibits OC differentiation and function *in vitro* and suppresses inflammatory bone erosions in mice [112–114]. Tregs negatively influence osteoclastogenesis through two mechanisms: the first involves cell contact, whereas the second is contact independent and involves the production of cytokines [115–118]. In particular, Kim et al. found that Tregs inhibit OC differentiation from peripheral blood mononuclear cells in a cytokine-dependent manner and proposed that TGF*β* and IL-4 cytokine secreted by Th2 cells may be the key cytokines responsible for the suppressive function of Tregs [115]. Recently, Tregs have been implicated in the mechanism by which estrogen suppresses OC differentiation and bone resorption through production of IL-10 and TGF*β*1 [116]. Another mechanism by which Tregs maintain control of immune function is by secretion of cytotoxic T-lymphocyte antigen 4 (CTLA4), an inhibitor that binds to CD80 and CD86 coreceptors on antigen-presenting cells and blocks their association with CD28 on T cells, thus dulling inflammatory responses [117]. However, direct antiosteoclastogenic effects of CTLA4 mediated on purified OC precursors were documented. The data suggest that in addition to an anti-inflammatory role of CTLA4, this receptor may directly suppress osteoclastogenesis by binding to CD80/CD86 on mononuclear OC precursors [119]. Interestingly, Tregs have also been shown to directly inhibit OC formation by CD11b monocytes treated with M-CSF and RANKL as well as to suppress resorption of pits *in vitro* by mature OCs.

### 4.3. CD8^+^ T Cells

CD8^+^ T cells have a protective role on bone. In particular, they profoundly suppressed osteoclastogenesis, mostly via soluble proteins. CD8^+^ T cells expressed a substantial amount of OPG along with RANKL [119]. However, blocking antibody to OPG did not reverse the suppression by CD8^+^ T cells, suggesting that other factor(s) is involved [119]. Anabolic PTH treatment in mice was found to significantly increase the production of Wnt10b by bone marrow CD8^+^ T cells leading to activation of canonical Wnt signaling in preosteoblasts. Demonstrating a key role of T cells in anabolic PTH action, T-cell-null mice displayed diminished Wnt signaling in pre-OBs and blunted osteoblastic commitment, proliferation, differentiation, and lifespan. These actions culminated in a diminished anabolic response in trabecular bone and a failure to increase bone strength. Furthermore, mice conditionally lacking Wnt10b production specifically in their T cells failed to induce an anabolic response to intermittent PTH [120]. Further studies involving conditional silencing of the PTH receptor specifically in T cells were found to blunt the capacity of intermittent PTH to induce T cell production of Wnt10b, thus abrogating activation of Wnt signaling in OBs, expansion of the osteoblastic pool, and increased BMD and trabecular bone volume in response to intermittent PTH. These data thus revealed a direct action of PTH on the T cell leading to Wnto10b production [120, 121]. Recently, an important role for CD8^+^ cells has been demonstrated in bone tumor burden protecting from bone metastasis [122].

#### 4.3.1. CD8 T REG

Although CD8 T REG have been documented in humans and mice [123–128], they have not been studied extensively, in part due to their low abundance (0.2 to 2% of CD8 T cells) in lymphoid organs. In comparison, the well-studied CD4 regulatory T cells, T_REG_, comprise 5–12% of CD4 T cell in the spleen. The FoxP3^+^ CD8 T cells and the T_REG_ have overlapping and distinct functions. Both cells express CD25 and the transcription factor, FoxP3 a marker of the regulatory T cells. The osteoclast-induced FoxP3^+^ CD8 T-cells secreted cytokines that could suppress formation and activity by OCs. The FoxP3^+^ CD8 T-cells did not affect the survival of OCs, but FoxP3^+^ CD8 T-cells could directly act on mature OCs to suppress actin ring formation. The ability of OCs to induce FoxP3^+^ CD8 T-cells and the ability of FoxP3^+^ CD8 T-cells to subsequently regulate OC function establishes a bidirectional regulatory loop between these two cells in the bone marrow. Notably, the regulatory loop does not require the presence, *in vitro*, of proinflammatory cytokines. Indeed, the ability to isolate functional FoxP3^+^ CD8 T-cells from mice, in the absence of any inflammatory disease, indicates that these cells have a role in maintaining skeletal homeostasis *in vivo* [129].

### 4.4. NK T Cells and *γδ* T Cells

#### 4.4.1. NK T Cells

Natural killer (NK) T cells are known to participate in the clearance of virus-infected, aberrant, or transformed cells [130]. Moreover, NK cells are poised for a rapid release of cytokines and growth factors that might influence the initiation and development of immune responses mediated by T and B cells [131–133]. Moreover, the activation of a particular subset of NK cells, the invariant NKT (iNKT) cells, increases OC development, maturation, and activity [134].

NK cells can be detected in the inflamed synovial tissue at an early stage of the disease, and they constitute up to 20% of all lymphocytes in the synovial fluid (SF) of patients with established RA [135, 136]. Recent evidence shows that this CD56bright NK cell subset has an upregulated expression of several chemokine receptors and adhesion molecules that may participate in its preferential recruitment into the inflamed synovium [137] and enable the cells to engage and subsequently activate monocytes through a variety of receptor-ligand interactions [135, 138, 139]. NK cells in the SF of RA patients efficiently trigger formation of OCs from monocytes. In particular, NK cells express both M-CSF and RANKL, which are responsible for osteoclastogenesis, and both molecules are further upregulated on NK cells by IL-15 [140, 141].

#### 4.4.2. *γδ* T Cells

Although the vast majority of circulating T-cells express *αβ* TCR chains, a subset of T-cells expresses a different TCR, containing a gamma (*γ*) chain paired with a delta (*δ*) chain, to form a *γδ* TCR heterodimer, and giving rise to a population of *γδ* T-cells. *γδ* T-cells represent only 1–10% of nucleated cells in the human peripheral circulation although their numbers are more abundant in tissues, in particular, epithelial tissues such as the skin, where *γδ* T-cells may represent the dominant T-cell population [142]. *γδ* T-cells are dissimilar to *αβ* T-cells in that their function is largely innate-like rather than adaptive and TCR specificity is directed almost exclusively towards nonpeptide antigens. They have been implicated in responses to inflammation, allergy, autoimmunity, infectious disease [142], and certain hematological tumors [142, 143]. They express growth factors important for tissue regeneration, such as fibroblast growth factor [142] and connective tissue growth factor, [144] that are critical for wound and skeletal fracture healing. Rather than representing a single population, *γδ* T-cells have been found to be quite heterogeneous. Although found only in humans and higher primates, V*γ*9V*δ*2 T-cells are a major subpopulation of *γδ* T-cells and are unique in their recognition of low-molecular-weight nonpeptide antigens. Recently, Kalyan et al. demonstrated that these unique innate T cells are lost in osteoporotic patients on amino-bisphosphonate treatment, and this loss is related to the potency of the systemic dose and the length of time on therapy and the diagnosis of osteonecrosis of the jaw [145, 146].

### 4.5. B Cells

In addition to this immune function, B cells have a close and multifaceted relationship with bone cells [147]. B cells differentiate from hematopoietic stem cells (HSCs) in supportive niches found on endosteal bone surfaces. Cells in the osteoblastic lineage sustain HSC and B cell differentiation in these niches. B cell differentiation is regulated, at least in part, by a series of transcription factors that function in a temporal manner. While these transcription factors are required for B cell differentiation, their loss causes deep changes in the bone phenotype. This is due, in part, to the close relationship between macrophage/OC and B cell differentiation. While the role of B cells during normal bone remodeling appears minimal, activated B cells play an important role in many inflammatory diseases with associated bony changes. In particular, B cells [148–150] and B-cell-derived plasma cells in multiple myeloma (MM) have been reported to have the potential to support osteoclastogenesis [151], possibly via direct expression of RANKL [152], decoy receptor 3 (DcR3) [153], or as an indirect consequence of IL-7 secretion [154, 155], a potent stimulator of bone resorption *in vivo* [156]. Malignant B-cell-derived plasma cells in MM produce also different cytokines inhibiting OB differentiation, such as sclerostin and DKK1 [151, 157, 158]. Moreover, B lymphopoiesis is stimulated during estrogen deficiency [159] while estrogen treatment downregulates B lymphopoiesis but upregulates immunoglobulin production [160]. B-lineage cells have consequently been suggested to play a role in ovariectomy-induced bone loss [156]. Interestingly, immature B cell populations expressing the marker B220 have been suggested to transdifferentiate along the OC pathway *in vitro* [161] providing a potential enhanced source of OC precursors and an explanation for a role of B-lineage cells in ovariectomy-induced bone loss. After the discovery of RANKL as the key osteoclastogenic cytokine, expression of this factor by B-lineage cells (B220+ cells, which in the bone marrow represent multiple populations of early B-cell precursors, immature B cells, and mature B cells) has been reported to be more abundant in ovariectomized mice than in sham-operated mice [162]. RANKL from B cells isolated from the bone marrow of estrogen-deficient postmenopausal women has been demonstrated to secrete RANKL [163], providing a plausible mechanism for a role of B cells in estrogen deficiency-bone loss. Peripheral blood B cells inhibit OC formation in a human *in vitro* model of osteoclastogenesis, in part by secretion of TGF*β*, [164] a cytokine that induces apoptosis of OCs [164–166] and that is reported to stimulate OPG production [167]. Depletion of B cells *in vivo* also aggravates bone loss in an animal model of periodontitis, suggesting that B cells may act to limit bone resorption under certain pathological conditions [168].

Recently, however, to better address this issue, Onal et al. made use of a state-of-the-art conditional B cell RANKL KO mouse, to reevaluate the role of mature B cells in ovariectomy-induced bone loss. This high-sensitivity model did indeed reveal a small contribution of mature B cells to ovariectomy-induced bone loss as mice lacking RANKL in B lymphocytes were partially protected from the increase in OC numbers and bone loss caused by ovariectomy in cancellous bone, although not in cortical bone, in the conditional KO mice [169]. 

The prominent role of B cells is also documented in an animal model of HIV-1 infection. In particular, it has been recognized as strong defect in skeletal homeostasis that led to a significant decline in bone mineral density and in bone volume. These alterations in skeletal mass were consistent with significantly elevated OC numbers and bone resorption, a consequence of a significant decline in B cell OPG production, compounded by a significant increase in B-cell production of RANKL. Production of RANKL is indeed an established property of activated B cells [170, 171] and of B-cell precursors [172]. This imbalance in the RANKL/OPG ratio was favorable to osteoclastic bone resorption and was likely further exacerbated by a dramatic increase in the number of OC precursors [173]. Clinical studies to ratify these changes in humans are currently underway.

Furthermore, B-cell to T-cell crosstalk may regulate B-cell production of bone-active cytokines, because B cells suppress osteoclastogenesis when activated by Th1 cytokines while promoting osteoclastogenesis when stimulated with Th2 cytokines [174]. *In vitro* ligation of the costimulatory molecule CD40 on human tonsil-derived B cells with an activating antibody is reported to stimulate B-cell OPG production [175]. Physiologically CD40 interacts with its cognate ligand, CD40 ligand (CD40L), a molecule expressed on activated T cells during antigen presentation by antigen-presenting cells such as B cells, macrophages, and dendritic cells [176], and acts in priming of naive CD8^+^ cells [177]. 

Moreover, both T cells and B cells are involved in the process of basal bone turnover. In addition to the well-documented roles of lymphocytes in bone destruction under pathological conditions, both T and B cells cooperate to play a critical role in limiting basal bone resorption *in vivo*. This protective effect is centered on a mechanism involving the production of OPG by B-lineage cells, and augmented by T cells, via CD40/CD40L costimulation [178].

### 4.6. Dendritic Cells

Dendritic cells (DCs) are highly differentiated antigen-presenting cells (APCs) that play a key role in the initiation and regulation of T cell immunity to pathogens and tumors while at the same time preventing immune responses against self-tissues or environmental antigens [179]. Under normal conditions, DCs are rarely localized in the bone proper or adjacent stroma, and they do not seem to contribute to bone remodeling, as DC-deficient animals have no skeletal defects [180]. On the other hand, it has been clearly documented that active lesions of rheumatoid arthritis and periodontitis harbour both mature and immature DC located in different compartments of the affected synovial and periodontal tissues surrounded by bone [181–186]. Interestingly, at active disease sites of rheumatoid arthritis and periodontitis, DCs can form aggregates with T cells in inflammatory foci, whereby they can interact through RANK-RANKL signaling *in vivo*, and they have been described as indirect players influencing inflammation-induced bone loss through regulating T cell activity [181–187].

Recently, Rivollier et al. [188] showed that human peripheral blood Mo-derived DCs can transdifferentiate into OCs in the presence of M-CSF and RANKL *in vitro*, suggesting that DCs might directly contribute to osteoclastogenesis. Alnaeeli et al. tested whether DC/T cell interactions can support DDOC development by *in vitro* cocultures using pure CD11c+CD11b−DC subset (lacking classical OC precursors [189, 190]) derived from total bone marrow (BM) cultures in the presence of granulocyte-macrophage colony-stimulating factor (GM-CSF) and interleukin IL-4 [191]. The results suggest that (1) murine CD11c+DC can develop into functional OCs (DDOCs) during immune interactions with CD4^+^ T cells and microbial products or protein Ags in the bone environment and (2) DDOCs can induce bone resorption after adoptive transfer onto NOD/SCID mouse calvarias *in vivo* [191]. These findings indicate a potentially critical contribution of CD11c+DC subset(s) to elevated osteoclastogenesis associated with inflammatory bone disorders where they act not only as potent APCs for immune activation and regulation but also as direct contributor to bone destruction. DCs also promote hyperactive osteoclastogenesis in MM bone disease [192, 193] because their number is higher within the erosive lacunae. In addition, they may undergo OC-like transdifferentiation following stimulation by the RANK-RANK-L [194]. Additionally, mature DCs may drive, within the tumor site, the expansion of a Th-17 clone leading to IL-17 overproduction that enhances osteoclastogenesis [195].

### 4.7. Neutrophils

Neutrophil granulocytes are the most abundant type of white blood cells in mammals and form an essential part of the innate immune system. Neutrophils are normally found in the blood stream. During the beginning (acute) phase of inflammation, particularly as a result of bacterial infection, environmental exposure [196] and some cancers [197, 198] neutrophils are one of the first responders of inflammatory cells to migrate towards the site of inflammation. The sites of bony lesions in humans and in animal models show massive infiltration of the prototypic inflammatory cells, neutrophils. Neutrophils are also implicated in human periodontitis [199], as well as several arthritis animal models [200–202]. Of note, although traditionally considered to be short-lived cells with limited synthetic capacity, activated neutrophils have been shown to synthesize considerable amounts of proteins and lipids that participate in the inflammatory process [203, 204]. In human neutrophils from inflammatory sites expressed high levels of RANKL [205]. Human, as well as murine, neutrophils strongly upregulate their expression of membrane RANKL after LPS stimulation and thus have the capacity to activate osteoclastic bone resorption through neutrophil-OC interactions [206]. The osteoclastogenic effect of neutrophil RANKL, demonstrated with human- and murine-activated neutrophils (purity > 95%), was reproduced with purified neutrophil membranes and fixed neutrophils, but not with culture supernatants of activated neutrophils in which no secreted RANKL was detected. Thus, RANKL expression in neutrophils differed from that in activated CD3^+^ lymphocytes, which express both cell surface and soluble RANKL [207, 208]. Moreover, neutrophils can affect OB functions in children on chronic glucocorticoid therapy as well as in tophaceous gout leading to decreased bone formation and increased bone resorption [209, 210].

## 5. Conclusion

Over the past two decades extraordinary advancement has been done in understanding the crosstalk between the bone and immune system in physiological and pathological conditions. Although numerous data arise from animal models, exciting data from human studies are emerging and as a consequence the first biological drugs targeting cytokines released from immune cells are emerging as alternative therapeutic management for inflammatory bone disease, such as arthritis and osteoporosis. However, despite the advancement made, further studies needed to elucidate the cross-talk between the bone and immune system.
